# Hypophysitis: Evaluation and Management

**DOI:** 10.1186/s40842-016-0034-8

**Published:** 2016-09-06

**Authors:** Alexander Faje

**Affiliations:** Neuroendocrine Unit, Massachusetts General Hospital and Harvard Medical School, 55 Fruit Street, Boston, MA 02114 USA

**Keywords:** Hypophysitis, Hypopituitarism, Diabetes insipidus

## Abstract

Hypophysitis is the acute or chronic inflammation of the pituitary gland. The spectrum of hypophysitis has expanded in recent years with the addition of two histologic subtypes and recognition as a complication of treatment with immune checkpoint inhibitors. Despite the increased number of published cases, the pathogenesis of hypophysitis is poorly understood, and treatment strategies are diverse and controversial. The diagnosis of hypophysitis generally requires histopathologic confirmation. The presentation and clinical course of hypophysitis varies. Hypophysitis can resolve spontaneously, relapse may occur, and some cases can be refractory to treatment.

## Background

Hypophysitis has gained greater clinical recognition over time. Several histologic variants and causative agents have been identified. Although hypophysitis remains a rare diagnosis, the number of published cases has increased substantially and expanded to involve a more gender and age diverse population. The quantity and quality of available information is limited, however, and consensus, especially regarding treatment, has been elusive. Prospective studies are necessary to better define optimal diagnostic and management strategies.

Hypophysitis can be classified according to etiology, morphology, and/or histopathology. Etiology refers to primary or secondary cases of hypophysitis. Primary hypophysitis refers to isolated inflammation of the pituitary not associated with medications, systemic inflammatory disorders, infections, or other diseases. Secondary hypophysitis includes cases associated with immunotherapy (interleukin 2, interferon, and medications targeting cytotoxic T-lymphocyte antigen-4 [CTLA-4] or programmed cell death 1 [PD-1]) [1–6], rupture of sellar cysts (Rathke’s cleft cysts and craniopharyngiomas), and rarely, pituitary adenomas [7–15]. Some authors utilize the term secondary hypophysitis more broadly and also include systemic inflammatory processes which may involve the pituitary gland (such as sarcoidosis, Wegener’s granulomatosis, Crohn’s disease, Takayasu’s arteritis, Cogan’s syndrome), inflammatory cell proliferative disorders (Langerhans cell histiocytosis [LCH] and Erdheim-Chester disease [ECD]), infections (tuberculosis, syphilis, Whipple’s disease, mycoses), and tumor-associated inflammatory infiltrate (germinoma).

Morphologic categorization is made according to whether inflammation involves the anterior pituitary gland (adenohypophysitis), posterior gland and stalk (infundibuloneurohypophystis), or entire gland (panhypophysitis).

Histologic subtypes of hypophysitis include the following: lymphocytic, granulomatous, xanthomatous, and plasmacytic (Table 1). Occasionally, mixed histology is encountered [16]. Necrotizing hypophysitis has also been proposed as an additional variant, but it has only been reported in 3 cases [17, 18]. Lymphocytic hypophysitis is characterized by diffuse lymphocyte infiltration (primarily T cells) of the pituitary gland. Lymphoid follicles can be observed and occasional plasma cells, eosinophils, and fibroblasts may also be present [19]. Granulomatous hypophysitis shows large numbers of multinucleated giant cells and histiocytes with granuloma formation [20, 21]. Xanthomatous hypophysitis demonstrates lipid-laden “foamy” histiocytes without the presence of granulomas [22, 23]. Plasmacytic hypophysitis, also termed IgG4-related hypophysitis, has extensive gland infiltration by plasma cells with a high degree of IgG4 positivity [24–26]. Pituitary gland fibrosis and atrophy may occur in later stages of these hypophysitis variants.

**Table 1:** Histologic subtypes of hypophysitis and patient characteristics

	Gender predominance	Association with pregnancy	Mean age of presentation
Lymphocytic	Female, ~3:1	Yes	4th decade
Granulomatous	Female, ~3:1	No	5th decade
Xanthomatous	Female, ~3:1	No	4th decade
Plasmacytic (IgG4-related)	Male, ~2:1	No	7th decade

Mixed histology is observed occasionally, and necrotizing hypophysitis has been proposed as an additional category. Data abstracted from references [20, 23, 25, 27, 66]

Precise usage of the term hypophysitis is important. Loose or inconsistent application (such as grouping germinoma-associated inflammation and primary lymphocytic hypophysitis) can cause reader confusion and suggest inappropriate treatments rather than provide diagnostic clarification. Unfortunately, such cases have been mixed with primary hypophysitis in some review paper data sets [27]. Unless otherwise stated, further discussion in this manuscript will focus on patients with primary hypophysitis. A caveat exists for IgG4-related hypophysitis, which is often a manifestation of systemic disease with involvement of multiple organs. Most authors have not grouped IgG4-related hypophysitis in the general category of secondary hypophysitis, though it may be reasonable to do so. This manuscript does include an examination of IgG4-related hypophysitis. Given the expanding applications of immune checkpoint inhibitors and increasing frequency of this form of secondary hypophysitis, brief discussion will also be devoted to immunotherapy-associated hypophysitis.

## Epidemiology

The annual incidence of hypophysitis is estimated to be 1 in 7–9 million. Hypophysitis accounts for approximately 0.4 % of pituitary surgery cases (based on a group of large surgical series totaling nearly 10,000 procedures at 5 centers) [28–32].

Lymphocytic hypophysitis was first reported in 1962 [33], and granulomatous hypophysitis was described in the early twentieth century [34, 35]. The first cases of xanthomatous hypophysitis and IgG4-related hypophysitis were published more recently in 1998 and 2004, respectively [22, 36].

Lymphocytic hypophysitis is the most common histologic variant, with over 390 cases reported. Granulomatous hypophysitis is the next most frequent subtype, followed by xanthomatous and IgG4-related hypophysitis [25]. Lymphocytic hypophysitis was initially thought to occur only in adult women, but cases were subsequently described in men [37] and children [38–40]. Lymphocytic hypophysitis does occur more frequently in women compared to men (approximately 3:1 ratio of cases [27]), in large part because of its association with pregnancy [41]. Though recent series have not shown as strong a relationship [42, 43], the majority of cases among reproductive-aged women appear to occur during the end of pregnancy or the first few months after delivery [16, 27]. The incidence of lymphocytic hypophysitis peaks during the fourth decade of life and is uncommon in children and the elderly.

Granulomatous and xanthomatous hypophysitis also occur more frequently in women (approximately 3:1 ratio of cases), but neither form is linked with pregnancy. Xanthomatous hypophysitis and lymphocytic hypophysitis have a similar mean age of presentation, but granulomatous hypophysitis is diagnosed more often at a slightly later timepoint in the fifth decade [20, 23]. IgG4-related hypophysitis occurs more frequently in men and tends to develop at a more advanced age in the seventh decade of life. IgG4-related hypophysitis also does not have an association with pregnancy [25, 26].

Immunotherapy-associated hypophysitis occurs in up to 10–15 % of patients receiving agents targeting CTLA-4, on average 2–3 months after starting therapy. Older age and male gender may be risk factors for the development of hypophysitis with anti-CTLA-4 medications. Hypophysitis is comparatively rare following treatment with anti-PD-1 agents. Hypophysitis has also been reported very rarely after treatment with interleukin 2 and interferon [6].

## Clinical presentation

Patients with hypophysitis present with symptoms related to mass effect from pituitary gland enlargement and pituitary/hypothalamic dysfunction. Headache is the most common presenting symptom, occurring in about half of patients. Visual symptoms due to compression of the optic nerves and/or cranial nerves III, IV, and VI in the cavernous sinuses can occur in a substantial minority of patients [16, 27, 42]. Cavernous carotid artery occlusion is a rare complication of hypophysitis [31, 44–46]. The onset of symptoms, including headache, can be insidious, subacute, or acute even mimicking apoplexy [42, 47, 48].

The majority of patients with hypophysitis have multiple anterior pituitary hormone deficiencies, and anterior panhypopituitarism is not uncommon. The severity of hormone deficiencies may appear to be out of proportion to radiographic findings. Serum prolactin levels may be low, normal, or elevated [16, 19, 27, 42]. Unlike what is observed in clinically nonfunctioning pituitary adenomas [49], there is not a clear hierarchy of anterior pituitary hormone deficiencies in hypophysitis patients. Hypothalamic pituitary adrenal axis dysfunction is frequently present. Diabetes insipidus is also common and may occur in up to half of patients [16, 19, 27, 42].

Immunotherapy-associated hypophysitis often presents with headache and anterior hypopituitarism. The degree of pituitary enlargement is typically mild, and compression of the optic apparatus is very rare. Unlike other forms of hypophysitis, diabetes insipidus is extremely unusual in patients with immunotherapy-associated hypophysitis [6].

## Diagnosis

The differential diagnosis for primary hypophysitis is broad, and ultimately histopathology (which is not always possible to obtain) is required for confirmation. Alternative diagnostic considerations include anatomic variants (a small/narrow sella turcica with specious pituitary enlargement) and congenital malformations, pituitary hyperplasia, solid and cystic sellar/suprasellar lesions (such as pituitary adenomas with or without apoplexy, Rathke’s cleft cyst, craniopharyngioma, pituitcyte-derived tumors, hamartoma, dermoid or epidermoid cyst, gangliocytoma, lipoma), malignancies (central nervous system germinoma, lymphoma, glioma, metastatic lesions, LCH, ECD), systemic inflammatory disorders (sarcoidosis, Wegener’s granulomatosis, Crohn’s disease, Takayasu’s arteritis, Cogan’s syndrome), and infections (tuberculosis, syphilis, Whipple’s disease, mycoses). A thorough evaluation is necessary to accurately diagnose hypophysitis, especially in the absence of tissue confirmation. In the largest series of pituitary stalk lesions published to date, only 4 % of pathology-proven diagnoses represented primary hypophysitis (and only one-third of cases were inflammatory disorders of any type). Significantly, more than half of the confirmed stalk lesions represented neoplastic processes, and half of these cases were metastatic lesions [50]. A positive response to glucocorticoids, often interpreted as supporting evidence for hypophysitis, is not specific for inflammatory processes. Glucocorticoids are part of standard treatment regimens for LCH and ECD [51, 52], and temporary treatment responses can be observed in lymphoma and intracranial germinomas [53–55]. Treatment responses in the latter are likely due to effects on tumor infiltrating lymphocytes. This lymphoid infiltrate can be significant enough that misdiagnosis can even occur after tissue biopsy due to sampling error [56–58].

Certain radiology findings may support a diagnosis of hypophysitis. These imaging characteristics include homogenous enhancement of the pituitary, diffuse symmetric gland enlargement, midline stalk thickening, absence of a posterior pituitary bright spot, normal sellar size, dural thickening, parasellar T2-weighted hypointensity, and parasellar mucosal thickening. One group described a radiologic scoring model with an apparent high ability to distinguish hypophysitis from pituitary adenomas [59]. This model was not assessed for its discriminatory value against other potential diagnoses. Ultimately, radiologic findings are not specific for hypophysitis, especially compared to nonadenomatous sellar lesions.

Diagnostic criteria have been proposed for IgG4-related hypophysitis. These include the following: 1) pituitary histopathology demonstrating mononuclear infiltration with greater than 10 IgG4-positive cells per high-powered field, 2) magnetic resonance imaging (MRI) showing a sellar mass and/or stalk thickening plus biopsy-proven IgG4-related disease at another tissue site, or 3) sellar mass and/or stalk thickening plus a serum IgG4 level > 140 mg/dl and a radiologic and clinical response to treatment with glucocorticoids [24]. These proposed criteria may be inadequate in some circumstances. Recent studies have shown that neither serum IgG4 levels [60, 61] nor IgG4-positive tissue staining [62–65] are necessarily sensitive nor specific for IgG4-related disease. According to more recent international consensus criteria, diagnoses of IgG4-related disease are primarily based upon pathology demonstrating 2 of 3 major histopathological features (dense lymphoplasmacytic infiltrate, storiform fibrosis [a cartwheel or whirled pattern of fibrosis, at least focally], and obliterative phlebitis) with appropriate clinicopathologic correlation. IgG4 serum levels and tissue staining have important secondary roles [66]. Published cases of IgG4-related hypophysitis typically do not comment on the presence or absence of such histologic features [26]. When it is described, storiform fibrosis has been reported in some [67] but not all cases of IgG4-related hypophysitis [68] following tissue analysis. Obliterative phlebitis has not been reported in any case of IgG4-related hypophysitis. As Ngaosuwan et al. noted, the diagnosis of IgG4-related hypophysitis is difficult without the presence of other organ involvement [69]. Although only a minority of reported IgG4-related hypophysitis cases have included histopathology, almost all patients had other organ involvement [70].

No case of immunotherapy-associated hypophysitis has been confirmed by pituitary gland biopsy. Diagnoses are established clinically based upon the close temporal relationship of immunotherapy treatment and the development of hypopituitarism with reversible pituitary enlargement [6, 71]. The relationship of lymphocytic hypophysitis with pregnancy [27, 41] may also allow a clinical diagnosis to be made with a reasonable degree of confidence in some pregnant or early postpartum women without tissue confirmation when appropriate imaging and biochemical findings are present with an otherwise negative thorough diagnostic evaluation. Patient demographics and coexistent medical conditions may also help focus diagnostic considerations. For example, intracranial germinomas have a peak incidence in the second decade of life and are extremely rare after the age of 30 [72]. LCH can be diagnosed at any age, but the incidence of this disease progressively declines throughout life [73, 74].

Given the broad differential diagnosis for hypophysitis, caution and close follow up is strongly advised for the treatment of presumed cases lacking histopathologic confirmation.

## Treatment

No prospective controlled studies have examined the treatment of hypophysitis, and a limited number of cases detail the natural history of untreated disease. Available retrospective data sets are confounded by reporting and treatment selection biases and likely encompass a heterogeneous group of diseases due to the lack of histologic confirmation in many cases and variable clinical evaluation. Medical therapies differ significantly by the type of agent, dosage, and duration of treatment.

**Fig. 1:**
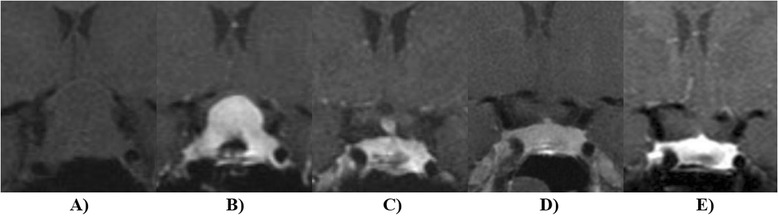
Hypophysitis was diagnosed in a 30 year old during the late third trimester of pregnancy. The patient presented with 3 weeks of progression vision loss. Panel **a** depicts a coronal pre-contrast T1-weighted image of the pituitary. Transsphenoidal biopsy (Panel **b**) demonstrated lymphocytic hypophysitis and glucocorticoid therapy was begun with prednisone 60 mg daily. Following delivery, the pituitary gland decreased in size (Panel **c**) and remained stable 2 months (Panel **d**) and 5 months (Panel **e**) after glucocorticoid taper and discontinuation

Symptoms from mass effect, such as optic nerve compression and other cranial nerve palsies, and severe headache are general indications for the treatment of hypophysitis (Fig. 1). Practice patterns vary for less clinically severe cases. It is unclear whether treatment with immune suppressing medications improves pituitary function outcomes compared to supportive therapy.

Spontaneous resolution of pituitary enlargement has been observed in a number of published cases of hypophysitis [19]. One group recently reported regression of radiologic findings in 15/15 patients receiving supportive therapy [43]. A large recent retrospective review of hypophysitis cases in Germany noted radiologic improvement or stability in 16/22 cases without active treatment [75]. Pituitary surgery (gross total resection or partial resection) and glucocorticoid therapy appeared to be somewhat more effective at mass reduction in that study. Surgery (generally patients undergoing gross total resection) was associated with less improvement and greater loss of pituitary function. Patients receiving glucocorticoid therapy had a significant risk of relapsing pituitary enlargement and experienced frequent side effects from treatment. Approximately one-quarter of patients receiving supportive therapy demonstrated improvement in pituitary function, and 82 % of that group had stable or improved function. Similar results were reported in the patients treated with pharmacologic doses of glucocorticoids. Headache resolution was similar in all three groups [75]. Comparable rates of pituitary function recovery were reported following supportive therapy by Khare et al. [43]. A review by Lupi et al. suggested that pituitary function improvement may occur in approximately one-half of patients treated with glucocorticoids. Importantly, histopathology was available in only 22 % of these patients [76]. Similarly, a minority of patients had tissue confirmation in the study from Germany. Moreover, evaluation for secondary causes of hypophysitis was limited in the majority of those patients [42, 75]. Limited clinical evaluations were also frequent in the largest review of granulomatous hypophysitis cases [20].

Other immunosuppressive agents such as methotrexate, azathioprine, rituximab, infliximab, cyclosporine, and mycophenolate mofetil have been utilized in a small number of patients with hypophysitis [75–82]. Treatment with stereotactic radiosurgery and fractionated radiotherapy has been reported in a few patients, typically with refractory disease. Radiation dosages ranged from low levels to higher amounts used to treat pituitary adenomas [31, 83, 84].

Patients with immunotherapy-associated hypophysitis have been treated with physiologic to high-dose glucocorticoids. Although it is unclear whether pharmacologic dosages of glucocorticoids improve patient outcomes, higher doses do not appear to negatively impact the antitumor efficacy of immunotherapy or patient survival [6]. Improvement of pituitary function occurs in some patients following the resolution of hypophysitis; thyroidal and gonadal axis normalization occurs more frequently than adrenal recovery. The development of hypophysitis may be associated with improved patient survival in melanoma patients treated with Ipilimumab [71, 85].

## Pathogenesis

The mechanisms underlying the development of hypophysitis are unknown. Other autoimmune diseases coexist in a portion of patients with hypophysitis. Unlike many of these other conditions, pituitary autoantigens in hypophysitis have not yet been clearly identified. Several candidates have been proposed, including growth hormone, pituitary gland specific factors 1a and 2 [86, 87], alpha-enolase and gamma-enolase [88, 89], secretogrannin II [90], chorionic somatomammotropin, CGI-99 [91], and corticotroph-specific transcription factor [92]. Measurements of antibodies to these proteins, however, do not have sufficient sensitivity and specificity to be diagnostically useful [16, 93]. Given the lack of clinically validated autoantigens, many studies have utilized indirect immunofluorescence (IIF) to detect the presence of pituitary autoantibodies. Ricciuti et al. systematically described methodologic limitations of IIF in the assessment of anti-pituitary antibodies, their potential effects on data interpretation, and methods to optimize results [94].

The pathogenic role of IgG4 in IgG4-related disease, including hypophysitis, is unclear, and it has been suggested that elevation of these antibodies may represent a bystander phenomenon [95]. IgG4 predominance often correlates with immune downregulation, in part due to its ability to participate in fragment antigen binding arm exchange [96].

Immunotherapy agents presumably can activate an autoimmune process directed against unidentified pituitary antigens. Pituitary autoantibodies were detected in patients who developed hypophysitis following treatment with Ipilimumab (a monoclonal antibody targeting CTLA-4), but these antibodies were not present in patients without hypophysitis. CTLA-4 is also expressed by the pituitary gland, and treatment with Ipilimumab may directly target pituitary cells via activation of the classical complement pathway and antibody-dependent cell-mediated cytotoxicity (ADCC) [97–99]. Pituitary CTLA-4 expression levels appear to vary widely [100] and may affect the risk of developing hypophysitis following treatment with Ipilimumab. In support of this hypothesis, hypophysitis has not been reported in patients with germline CTLA-4 mutations, although many of these patients had other severe autoimmune diseases which can occur following treatment with Ipilimumab [101, 102]. It is unknown whether PD-1 is expressed by the pituitary gland. Notably, anti-PD-1 agents are IgG4-based antibodies, which can not activate the classical complement pathway and are not effective mediators for ADCC [96, 103–105].

## Conclusions

Currently, the diagnosis of primary hypophysitis typically requires a thorough evaluation for other potential neoplastic lesions, infiltrative diseases, infection, and systemic inflammatory processes plus histopathologic confirmation. In some cases, tissue biopsy may not be feasible. Cases of immunotherapy-associated hypophysitis and lymphocytic hypophysitis associated with pregnancy may potentially be diagnosed with some degree of confidence without surgery. Cranial nerve deficits due to mass effect from pituitary gland enlargement and severe headache are general indications for treatment with medical therapy and/or surgery. It is unclear whether active treatment improves clinical outcomes compared to supportive therapy for more mild cases of hypophysitis, and the therapies may be associated with side effects. Pituitary gland debulking rather than gross total resection is more commonly performed. Glucocorticoids (at variable dosages and duration) are the most frequent choice for medical therapy, though many other immunosuppressive agents have been utilized in the treatment of hypophysitis. Even when treatment is initially successful, disease recurrence is not uncommon. Radiation therapy appears promising, especially for refractory cases of hypophysitis, but the available published data consists of only a handful of patients.

Hypophysitis is an increasingly recognized but rare and poorly understood heterogeneous disease. The pathogenesis of primary hypophysitis is not yet known, and clinically validated disease markers have not been identified. In the absence of more detailed knowledge, various etiologic, morphologic, and histologic categories have been proposed, but the clinical utility of such schemas is limited. Inconsistent usage of terminology and variable diagnostic evaluations have also clouded data interpretation. Available studies are largely limited to retrospective series that likely include patients with diverse pathologies. Clinical investigation is constrained by the rarity of the disease. The acquisition of sufficient controlled prospective data will require multicenter collaboration. Until such investigations take place, optimal management strategies will remain largely undefined and controversial.

## Abbreviations

ADCC: Antibody-dependent cell-mediated cytotoxicity
CTLA-4: Cytotoxic T-lymphocyte antigen-4
ECD: Erdheim-Chester disease
IIF: Indirect immunofluorescence
LCH: Langerhans cell histiocytosis
MRI: Magnetic resonance imaging
PD-1: Programmed cell death 1
